# Comparison of Interrupted and Uninterrupted Anticoagulation Therapy for Patients With Atrial Fibrillation Undergoing Catheter Ablation: A Meta-Analysis

**DOI:** 10.7759/cureus.30742

**Published:** 2022-10-27

**Authors:** Sidra Jamil, Saima Batool, Saad Ehsan Ullah, Yared N Aschalew, Tafseer Zahra, Leladher Maheshwari, Venkata Anirudh Chunchu, Adil Amin

**Affiliations:** 1 Accident and Emergency, Glan Clwyd Hospital, Rhyl, GBR; 2 Internal Medicine, Hameed Latif Hospital, Lahore, PAK; 3 Internal Medicine, Shaikh Zayed Hospital, Lahore, PAK; 4 Internal Medicine, Inova Alexandria Hospital, Alexandria, USA; 5 Medicine, California Institute of Behavioral Neurosciences & Psychology, Fairfield, USA; 6 Medical College, Bahria University Health Sciences, Karachi, PAK; 7 Medical College, Avalon University School of Medicine, Willemstad, CUW; 8 Cardiology, Pakistan Navy Station Shifa, Karachi, PAK

**Keywords:** thromboembolic events, non valvular atrial fibrillation, atrial fibrillation, catheter ablation, uninterrupted anticoagulant, interrupted anticoagulant

## Abstract

Adequate periprocedural anticoagulation is important to prevent complications like transient ischemic attack, stroke, severe esophageal injury, and pulmonary vein stenosis. The aim of this meta-analysis was to compare uninterrupted anticoagulation therapy with interrupted anticoagulation therapy for patients with arrhythmias undergoing catheter ablation. The current meta-analysis followed the Preferred Reporting Items for Systematic Reviews and Meta-Analyses (PRISMA) guidelines. Several online databases were searched, such as PubMed, Cochrane Library, and Embase, to search for relevant randomized controlled trials (RCTs). The primary outcome included thromboembolic events. Secondary outcomes included major bleeding events and minor bleeding events. A total of eight RCTs were included in the current meta-analysis, encompassing a total of 3893 patients. No significant differences were reported in relation to thromboembolic events (RR: 2.39, 95% CI: 0.41-13.97, p-value: 0.33), major bleeding events (RR: 0.99, 95% CI: 0.50-1.96, p-value: 0.98) and minor bleeding events (RR: 1.55, 95% CI: 0.56-4.30, p-value: 0.40) between the two study groups. This meta-analysis did not find any conclusive evidence for the absence of any difference between the two strategies.

## Introduction and background

Atrial fibrillation is one of the most common arrhythmias in adults, and its prevalence is predicted to be increased significantly during the next two to three decades [1]. Rhythm and rate control can reduce symptoms of atrial fibrillation and preserve cardiac function. However, studies conducted in the past failed to show a clear advantage in relation to long-term mortality or morbidity [2]. Catheter ablation is a safe, well-established, and efficient strategy to achieve rhythm control in individuals with atrial fibrillation who are either refractory to pharmacologic rhythm control or intolerant to pharmacologic rhythm control [3]. In patients with persistent or paroxysmal atrial fibrillation without significant risk factors for atrial fibrillation recurrence, the ESC's 2020 guidelines advise catheter ablation for pulmonary vein isolation (PVI) for control of rhythm after pharmacological therapy fails to relieve symptoms of occurrence of atrial fibrillation [4].

Previous studies have demonstrated that nearly 5-7% of patients undergoing atrial fibrillation catheter ablation experience periprocedural complications [4-5]. Some of these complications can be life-threatening, including transient ischemic attack, stroke, severe esophageal injury, and pulmonary vein stenosis [5]. To prevent these complications, adequate periprocedural anticoagulation is important. Previous studies have shown that uninterrupted vitamin K antagonist (VKA) therapy is effective in preventing bleeding and thromboembolic complications. However, studies have also shown that direct oral anticoagulants (DOAC) have similar safety and efficacy profiles compared to VKA in relation to catheter ablation [6-7].

During catheter ablation, the continuation of oral anticoagulation therapy presents a decision that needs to consider the procedure and the risk of thromboembolic and bleeding events [8]. Thus, effective management of oral anticoagulants is significant in reducing the risk of thrombosis and bleeding in atrial fibrillation patients undergoing catheter ablation [9]. In clinical practice, two strategies are being used that include interrupted or minimally interrupted anticoagulant therapy and uninterrupted oral anticoagulant therapy [10]. In the first one, oral anticoagulant therapy is minimally interrupted or completely interrupted that is defined as withholding up to two anticoagulant doses before the start of the procedure with or without the utilization of bridging therapy with subcutaneous low molecular weight heparin (LMWH) or intravenous heparin [11]. In the latter one, no reduction or interruption of oral anticoagulant therapy is done [12]. In recent years, new randomized controlled trials (RCTs) have been carried out that compared the efficacy and safety of uninterrupted versus interrupted oral anticoagulant therapy around procedures like catheter ablation [13-20]. However, the heterogeneity of currently used oral anticoagulant strategies in patients with atrial fibrillation undergoing catheter ablation focuses on the requirement for consensus recommendations and further data. Aggregate data from RCTs on oral anticoagulants may result in the detection of significant differences in groups than can aid in guiding healthcare professionals. Therefore, the current meta-analysis was conducted to compare uninterrupted anticoagulation therapy with interrupted anticoagulation therapy for patients with arrhythmias undergoing catheter ablation.

## Review

Methodology

The current meta-analysis was conducted by following the Preferred Reporting Items for Systematic Reviews and Meta-Analyses (PRISMA) guidelines.

Data Sources and Search Strategy

Several online databases were searched such as PubMed, Cochrane Library, and Embase to search for relevant randomized controlled trials (RCTs). Searching was performed from inception to 25th September 2022 without putting restrictions on language and year of publication. The following key terms and Medical Subject Headings (MeSH) terms were selected: “interrupted oral anticoagulant”, “uninterrupted oral anticoagulant”, “randomized control trial”, “atrial fibrillation”, and “catheter ablation”. A reference list of all included RCTs was also inspected.

Selection Criteria

In the current meta-analysis, we included RCTs that compared interrupted anticoagulant and uninterrupted anticoagulant therapy in adult patients with atrial fibrillation undergoing catheter ablation. We included all comparisons of uninterrupted and interrupted oral anticoagulants, including any modalities of interruption (minimally interrupted and completely interrupted). We excluded observational studies, non-randomized studies, case reports, and case series. The primary outcome included thromboembolic events. Thromboembolic events were assessed based on clinical parameters. Secondary outcomes included major bleeding events and minor bleeding events. These outcomes were defined by each RCT.

Study Selection and Data Extraction Process

The selection of studies was performed by two authors independently. Firstly, the abstract and titles of all publications retrieved in the electronic search were reviewed, followed by the full-text screening of potentially relevant studies. In case of any disagreement between the two authors, the issues were resolved through discussion and the involvement of the third author. Data extraction was done by two authors independently using a predesigned form. Data extracted from the selected studies included the name of the author, year of publication, sample size, characteristics of patients, and outcomes. Data extraction forms of both authors were compared, and any disagreement was resolved by discussion. Data were entered into RevMan Software.

Quality Assessment

The risk of bias assessment of all included studies was performed by two independent authors using the Cochrane risk of bias assessment tool. For each study, six domains were assessed including selection bias, detection bias, performance bias, reporting bias, attrition bias, and other potential sources of bias.

Data Synthesis and Analysis

The analysis was performed using Review Manager (RevMan) version 5.4.0 (Cochrane, London, United Kingdom). Risk ratio (RR) was computed for each outcome along with a 95% confidence interval (CI) using Mantel-Hanszel random effect model and presented using forest plots. A RR>1 shows an elevated risk of outcome in an interrupted oral anticoagulant group, whereas a RR<1 shows a higher risk in the uninterrupted oral anticoagulant group. A p-value of less than 0.05 was considered significant. Statistical heterogeneity was assessed using the I-square statistics, and a p-value less than 0.1 showed significant heterogeneity.

Results

**Figure 1:**
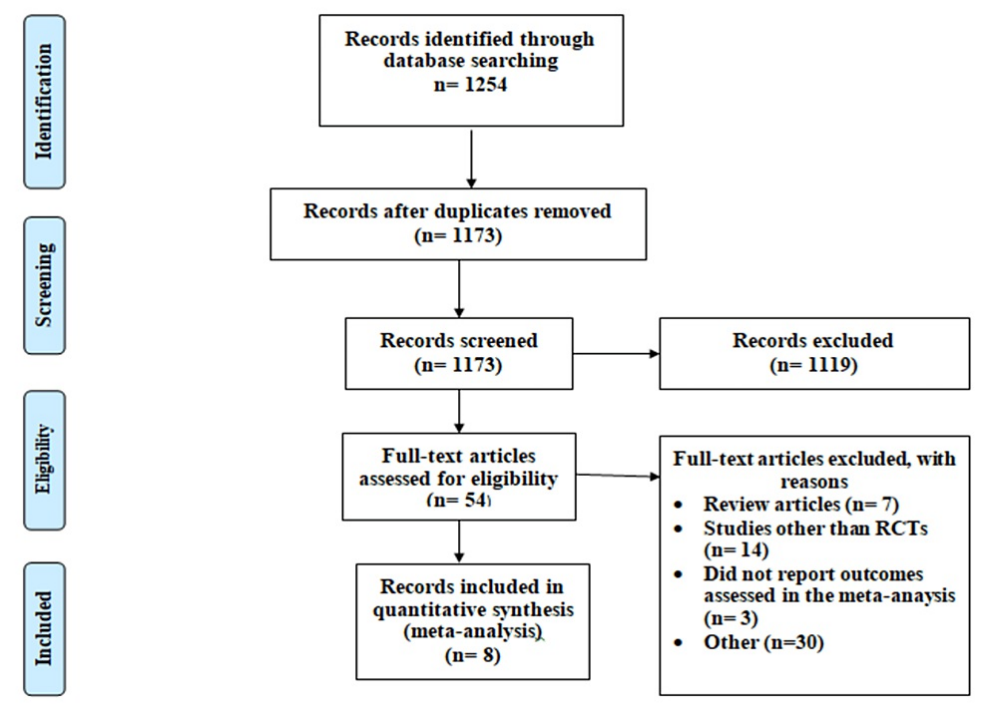
PRISMA flowchart of selection of studies

Figure 1 shows the PRISMA flowchart of the selection of studies. The online database search yielded 1254 articles. After removing duplicates, abstract and title screening were done of 1173 articles. Fifty-four articles remained. Their full text was retrieved and assessed for eligibility criteria. A total of eight RCTs were included in the current meta-analysis, encompassing a total of 3893 patients [13-20]. Characteristics of the included studies are shown in Table 1. The mean age of patients ranged from 58.3 to 70 years. The percentage of male patients ranged from 63.0% to 81.9%.

**Table 1:** Characteristics of the included studies DOAC: direct oral anticoagulant; VKA: vitamin K antagonist

First author	Year	Study setting	Groups	Type of interruption	Type of anticoagulant	Sample size	Mean age in years	Males (%)
Ando et al. [13]	2019	Single center	Interrupted anticoagulant	Minimally interrupted	DOAC	65	66.8	77.3
			Uninterrupted anticoagulant		DOAC	32		
Biase et al. [14]	2014	Multicenter	Interrupted anticoagulant	Completely interrupted	VKA	790	61.5	75.3
			Uninterrupted anticoagulant		VKA	794		
Nagao et al. [15]	2019	Single center	Interrupted anticoagulant	Minimally interrupted	DOAC	100	70	63
			Uninterrupted anticoagulant		DOAC	100		
Nakamura et al. [16]	2019	Single center	Interrupted anticoagulant	Minimally interrupted	DOAC	423	65	70.6
			Uninterrupted anticoagulant		DOAC	421		
Nogami et al. [17]	2019	Multicenter	Interrupted anticoagulant	Minimally interrupted	DOAC	220	65.5	74.9
			Uninterrupted anticoagulant		VKA	222		
Reynolds et al. [18]	2018	Multicenter	Interrupted anticoagulant	Minimally interrupted	DOAC	145	63.5	67.1
			Uninterrupted anticoagulant		DOAC	150		
Yoshimura et al. [19]	2017	Single center	Interuppted anticoagulant	Minimally interrupted	DOAC	50	58.9	81.9
			Uninterrupted anticoagulant		DOAC	55		
Yu et al. [20]	2019	Multicenter	Interuppted anticoagulant	Minimally interrupted	DOAC	220	58.3	74.5
			Uninterrupted anticoagulant		DOAC	106		

Risk of Bias Assessment

**Figure 2:**
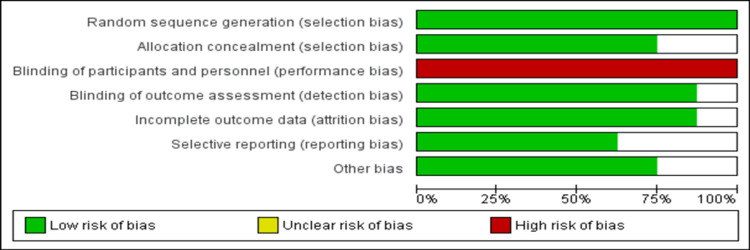
Risk of bias assessment

Figure 2 presents the overall risk of bias assessment of all included studies. The overall quality of the meta-analysis was moderate.

Risk of Thromboembolic Events

**Figure 3:**
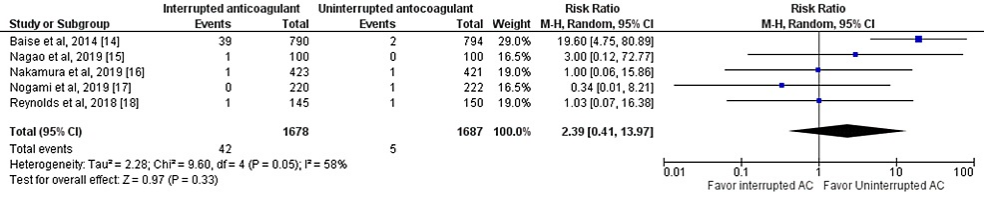
Forest plot of thromboembolic events Sources: [14-18] AC: anticoagulant

Five studies reported thromboembolic events between interrupted and uninterrupted anticoagulant therapy in patients with atrial fibrillation undergoing catheter ablation [14-18]. The pooled incidence of thromboembolic events was 1.39%. The incidence of thromboembolic events was higher in patients receiving interrupted anticoagulants (2.50%) compared to patients receiving uninterrupted anticoagulants (0.29%). However, no significant differences were reported between the two groups (RR: 2.39, 95% CI: 0.41-13.97, p-value: 0.33), as shown in Figure 3. Significant heterogeneity was reported between the study results (I-square: 58%, p-value: 0.05).

Risk of Major Bleeding Events

**Figure 4:**
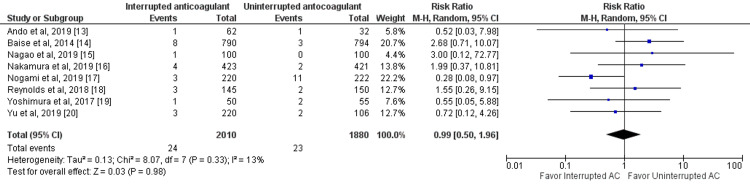
Forest plot of major bleeding events Sources: [13-20] AC: anticoagulant

All included studies compared major bleeding events between interrupted and uninterrupted anticoagulant therapy in patients with atrial fibrillation undergoing catheter ablation [12-20]. No significant differences were reported in relation to major bleeding events between the two study groups (RR: 0.99, 95% CI: 0.50-1.96, p-value: 0.98), as shown in Figure 4. There was no significant heterogeneity between the study results (I-square: 13%, p-value: 0.33).

Risk of Minor Bleeding Events

**Figure 5:**
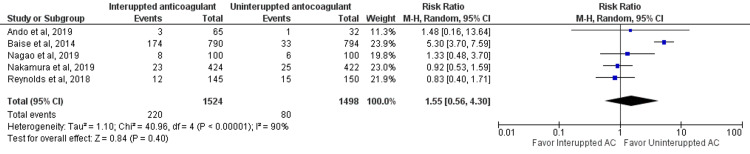
Forest plot of minor bleeding events Sources: [13-16, 18] AC: anticoagulant

Five studies assessed minor bleeding events between interrupted and uninterrupted anticoagulant therapy. No significant difference was reported between the two groups in relation to minor bleeding events (RR: 1.55, 95% CI: 0.56-4.30, p-value: 0.40), as shown in Figure 5. Significant heterogeneity was reported between the study results (I-square: 90%, p-value: 0.001).

Subgroup Analysis

Table 2 shows the findings of the subgroup analysis. Of the five studies that assessed thromboembolic events, one study used VKA in both arms, three studies utilized DOAC in both arms, and one RCT used VKA in one arm and DOAC in the other arm. We compared these three subgroups, and significant differences were found between the subgroups (p-value: 0.01).

Of the eight studies that compared major bleeding events between interrupted and uninterrupted anticoagulant therapy, one study used VKA in both arms; six studies utilized DOAC in both arms, and one RCT used VKA in one arm and DOAC in the other arm. We compared these three subgroups, and subgroup analysis showed significant differences between the sub-groups (p-value: 0.04).

**Table 2:** Findings of subgroup analysis DOAC: direct oral anticoagulant; VKA: vitamin oral anticoagulant; RR: risk ratio; CI: confidence interval * Significant at p-value<0.05

Outcomes	Subgroups	Number of studies	RR	95% CI	I-square
Thromboembolic event	VKA vs. VKA	1	19.6	4.75-80.89*	NA
	VKA vs. DOAC	1	0.34	0.01-8.21	NA
	DOAC vs. DOAC	6	1.36	0.26-7.23	0%
Major bleeding event	VKA vs. VKA	1	2.68	0.71-10.07	NA
	VKA vs. DOAC	1	0.28	0.08-0.97*	NA
	DOAC vs. DOAC	3	1.15	0.49-2.67	0%

Discussion

This meta-analysis was conducted to compare uninterrupted anticoagulation therapy with interrupted anticoagulation therapy for patients with arrhythmias undergoing catheter ablation. No significant difference was found between uninterrupted and interrupted anticoagulation therapy in terms of thromboembolic events, major bleeding events, and minor bleeding events. Ottoffy et al. discussed pooled analysis from RCTs, and no difference was found between uninterrupted and interrupted strategies in thromboembolic events [21]. However, the study demonstrated that the risk of major bleeding events was lower in the uninterrupted anticoagulant group. In the meta-analysis conducted by Ottoffy et al. only single strategy studies were included, which we excluded from the current meta-analysis [21]. Another meta-analysis conducted by Mao et al. included six RCTs and observational studies. Findings showed that no significant differences were reported between the two groups in relation to major bleeding events [22].

One of the major complications of atrial fibrillation is thromboembolic events. The low incidence of thromboembolic events with uninterrupted and interrupted anticoagulant therapy reported in the previous meta-analysis is reassured in the current meta-analysis.

Regarding thromboembolic events, Biase et al. [14] was the only RCT that used complete interruption. It means that warfarin was held two to three days before ablation and bridging with heparin. On the other hand, other studies used minimal interruption by skipping one or more than one dose of the anticoagulant with or without bridging of heparin. Biase et al. [14] was the largest study with the most number of thromboembolic events, while other studies reported no or fewer events. This is the only study in the current meta-analysis that showed that the risk of thromboembolic events is higher interrupted anticoagulant group compared to the uninterrupted anticoagulant group [14]. It enrolled large numbers of patients compared to other RCTs [13, 15-20].

Healthcare professionals experience dilemmas when deciding to continue or withhold oral anticoagulants for individuals with atrial fibrillation having catheter ablation procedures [23]. Interruption of oral anticoagulants, even though recommended by different guidelines, has been linked with negative outcomes, including an increased risk of thromboembolism, particularly when heparin bridging is utilized [24]. These complications may cause an increased stay in the hospital and an enhanced infection risk [25]. Thus, uninterrupted anticoagulant therapy has been preferred, with evidence suggesting this to be a feasible and safe option [24]. Theoretically, a brief pause in anticoagulant consumption could lessen anticoagulant activity right before the procedure and lessen the risk of bleeding issues. While point estimates of odd ratios for thromboembolic complications were in the reverse direction, meta-analyses on outcomes following interruptions of anticoagulants regimens were ambiguous and indicated trends towards roughly 20% lower major bleeding rates [26].

In individuals with atrial fibrillation undergoing catheter ablation, certain evidence support one strategy over another strategy. We were unable to offer the medical community a strong recommendation due to this ambiguity. However, a detailed examination of the kinds of anticoagulants utilized and how these medications were discontinued is merited. A completely interrupted strategy was linked to a lower likelihood of pre-procedural thromboembolism without enhancing the likelihood of major bleeding events, especially in individuals with long-standing persistent atrial fibrillation, according to the largest study utilizing vitamin K antagonists that contributed the most events to this meta-analysis. For RCTs that used minimal interruption and skipping doses of anticoagulants, the low rate of major bleeding and thromboembolism events can be accredited to the strategic nature.

The current meta-analysis has certain limitations. Firstly, the heterogeneity observed in the study outcomes could have limited the findings of this meta-analysis. We did subgroup analysis to explore the heterogeneity where we stratified groups by the type of anticoagulant yielded varied results for study outcomes. However, the findings of this meta-analysis should be interpreted with caution because of the smaller sample size of different meta-analyses and the availability of too few studies in each subgroup arm. Moreover, several studies have less than five events, and those estimates are most unreliable. In the future, more randomized control trials are required with large sample sizes to enhance the power of the findings, which will make them more generalizable.

## Conclusions

In conclusion, the meta-analysis found that the risk of thromboembolic, major bleeding, and minor bleeding events was not significantly different between patients who received interrupted anticoagulant therapy and uninterrupted anticoagulant therapy. However, due to the low sample size in the majority of studies included in this meta-analysis, the findings need to be interpreted with caution. In the future, multi-centered RCTs need to be conducted that include a larger sample size that can provide more precise findings helping healthcare professionals to select the best treatment option.
